# Non-Typhoidal *Salmonella* and the Risk of Kawasaki Disease: A Nationwide Population-Based Cohort Study

**DOI:** 10.3389/fimmu.2021.701409

**Published:** 2021-06-16

**Authors:** Thomas Yen-Ting Chen, Mei-Chia Chou, Jung-Nien Lai, Lu-Ting Chiu, Renin Chang, Yao-Min Hung, James Cheng-Chung Wei

**Affiliations:** ^1^ Department of Medical Research & Education, Kaohsiung Veterans General Hospital, Kaohsiung, Taiwan; ^2^ Department of Recreation and Sports Management, Tajen University, Pingtung, Taiwan; ^3^ Department of Physical Medicine and Rehabilitation, Kaohsiung Veterans General Hospital, Pingtung Branch, Pingtung County, Taiwan; ^4^ School of Chinese Medicine, College of Chinese Medicine, China Medical University, Taichung, Taiwan; ^5^ Department of Chinese Medicine, China Medical University Hospital, Taichung, Taiwan; ^6^ College of Medicine, China Medical University, Taichung, Taiwan; ^7^ Management Office for Health Data, China Medical University Hospital, Taichung, Taiwan; ^8^ Department of Emergency Medicine, Kaohsiung Veterans General Hospital, Kaohsiung, Taiwan; ^9^ Institute of Medicine, Chung Shan Medical University, Taichung, Taiwan; ^10^ College of Health and Nursing, Meiho University, Pingtung, Taiwan; ^11^ Department of Internal Medicine, Kaohsiung Municipal United Hospital, Kaohsiung, Taiwan; ^12^ School of Medicine, National Yang Ming University, Taipei, Taiwan; ^13^ Division of Allergy, Immunology and Rheumatology, Chung Shan Medical University Hospital, Taichung, Taiwan; ^14^ Graduate Institute of Integrated Medicine, China Medical University, Taichung, Taiwan

**Keywords:** Cohort study, Non-typhoidal *Salmonella*, Kawasaki disease, NHIRD, Taiwan

## Abstract

**Objective:**

The aim of this study was to investigate the relationship between non-typhoidal *Salmonella* (NTS) infection and the risk of Kawasaki disease (KD) by using a nationwide population-based data set in Taiwan.

**Methods:**

In this retrospective cohort study, we enrolled 69,116 patients under 18 years of age, with NTS from January 1^st^, 2000, to December 31^st^, 2013, using the population-based National Health Insurance Research Database of Taiwan. A comparison group without NTS was matched (at a 1:4 ratio) by propensity score. The two cohorts were followed from the initial diagnosis of NTS until the date of KD development or December 31^st^, 2013. Cox proportional hazard regression analysis was conducted to estimate hazard ratios (HRs) and 95% confidence intervals (CIs) after adjusting for covariates. Also, we conducted sensitivity analyses to examine our findings.

**Results:**

After adjusting for covariates, the risk of KD for the children with NTS was significantly higher than that of the comparison group (hazard ratio = 1.31; 95% confidence interval = 1.03-1.66; p < 0.01). Stratified analysis showed that the associated risk of the investigated outcome was significant in children aged ≤2 years (aHR= 1.31, 95% C.I. 1.02-1.69), in female patients (aHR= 1.46, 95% C.I. 1.03-2.08), and in those without allergic diseases.

**Conclusions:**

NTS is associated with an increased risk of KD in Taiwanese children.

## Introduction

Kawasaki disease (KD), which was previously called mucocutaneous lymph node syndrome, is an acute, febrile, inflammatory vasculitis of medium-sized muscular arteries with a definitive etiology that remains unknown (1, 2). KD is one of the most common vasculitides among children and especially occurs under 5 years of age (3). The prevalence of KD is highest in children of East Asian or Asian descent, particularly Japan, and lowest in those of European ancestry (4, 5). In Taiwan, the incidence of KD in children <5 years of age is reported to be the third highest in the world (5), immediately after Japan and Korea (6). It was also reported that the incidence of KD in Taiwan has increased in recent years, with a seasonal peak during April–June (7).

The clinical manifestations of KD can affect a variety of organs, with primary involvement of medium-sized muscular arteries, particularly the coronary arteries (3). According to the criteria established by Tomisaku Kawasaki, KD diagnosis is made based on a prolonged fever for more than 5 days, which is evidence of systemic inflammation, along with at least four of the five major clinical signs of mucocutaneous inflammation (8, 9). KD is typically a self-limited disease; however, it can have serious complications, with coronary artery lesions being the most severe ones, including coronary artery aneurysms and myocardial infarction, which might lead to morbidity and mortality (10). Although the cause of KD remains unknown, infection has been proposed as one of the various theories based on pathological and epidemiological evidence (11). Indeed, previous case series studies have reported localized KD outbreaks associated with various bacterial or viral pathogens (12, 13), and a retrospective review pointed out that infections are common at diagnosis of KD, with a wide variety of infectious agents found (14).

*Salmonella* bacteria are gram-negative, facultatively anaerobic members of Enterobacteriaceae that infect or colonize a broad range of mammalian hosts (15). They cause a variety of characteristic clinical infections in humans, such as gastroenteritis, enteric fever, and bacteremia. Enteric fever (typhoid fever) is caused by *Salmonella Typhi* and *Salmonella Paratyphi*; the other *Salmonella* serotypes are together referred to as non-typhoidal *Salmonella* (NTS), including *Salmonella serovars* Typhimurium and Enteritidis. NTS is a major cause of diarrhea and represent a global burden in both developing and developed countries, with NTS gastroenteritis being particularly prevalent in East Asia. It has been estimated that approximately 94 million cases of NTS gastroenteritis occur globally each year, and the incidence in East Asia is estimated to be as high as 4 cases per 100 persons annually (16).

Case reports exploring the linkage between NTS infection and vasculitis have been published. For example, Tavares et al. proposed NTS infection as a possible trigger for the development of polyarteritis nodosa, a systemic necrotizing vasculitis (17). Additionally, Filiz et al. reported a case of an interleukin 12 receptor beta 1 (IL-12Rβ1) deficiency with cutaneous leukocytoclastic vasculitis due to *Salmonella* enteritidis infection (18). As there have been no studies on the epidemiological relationship between NTS and subsequent development of KD, we conducted this longitudinal cohort study by using the Taiwan National Health Insurance Research Database (NHIRD) with the aim of exploring this possible link.

## Materials and Methods

### Data Source

We utilized the data from the National Health Insurance Research Database (NHIRD) of Taiwan, which collects comprehensive health care data for more than 99% of Taiwan’s population. The database of this program consists of registration files and claims data for reimbursement, including demographics, medical visits of all types, laboratory test codes, prescription codes, procedure codes, and diagnostic codes based on the International Classification of Diseases, 9th Revision, Clinical Modification (ICD-9-CM) system. In this study, we retrieved data from the Longitudinal Health Insurance Database 2000 (LHID 2000), a subset comprising the registration files and claim data of 1 million people randomly sampled from the registry in the year 2000 and covering the data of this group of people from 1995 to 2013. The LHID 2000 has been validated by the National Health Research Institutes (NHRI) as being representative of the national population in Taiwan, with no statistically significant differences in the distribution of age, sex, or health care costs between the dataset and the original NHIRD. Additionally, the accuracy of the NHIRD has been validated (19) as a resource for population research. To protect privacy, all identifiable information for each individual in the LHID 2000 was encrypted by the NHRI. This study was approved by the Institutional Review Board of China Medical University (CMUH104-REC2-115(AR-4)).

### Study Cohorts

In this retrospective cohort study, we identified 69,116 patients who were newly diagnosed with NTS (ICD-9-CM 003.xx) from the 1 million sampling cohort dataset, including both ambulatory and inpatient records, between January 1, 2000, and December 31^st^, 2013. The index date was defined as the date on which a diagnosis of NTS was initially made. To avoid coding errors in the claims data, we identified only patients who had at least two clinic visits or one admission with the diagnosis of nontyphoidal salmonellosis. A comparison group of non-NTS patients was also randomly selected from LHID 2000, matched at a ratio of 1:4, for age, sex, comorbidities and index dates.

Patients with NTS and those in the comparison group with a history of KD (ICD-9-CM 446.1) before the index date were excluded. A total of 276,464 subjects were enrolled as a comparison cohort (non-NTS group) in this study.

### Study Outcome and Confounders

We identified the KD as the outcome of our study, based on the diagnosis of ICD-9-CM code 446.1 (acute febrile mucocutaneous lymph node syndrome). To ensure the diagnostic validity, we defined patients with at least two clinic visits or one admission that were registered with ICD-9-CM code 446.1 in our main model (detailed in **Tables 1**–**3**). Moreover, we identified the KD patients who also had ICD-9-CM coded by pediatric specialists in the sensitivity analysis (model 2) (**Table 4**). In model 3 (**Table 5**), we further identified those KD patients with diagnosis on the Registry for Catastrophic Illness Patient Database, which includes selected serious injuries and illnesses. The KD diagnosis had been confirmed and certificated by a board-certified specialist in that database, and the application is further reviewed and approved by the NHI Bureau.

**Table 1:** Baseline characteristics of patients with and without non-typhoidal *Salmonella* (NTS) infection.

	Before PS matched					After PS matched				
	NTS					Non-NTS				
	No (n=23551857)		Yes (n=73837)		SMD	No (n=276464)		Yes (n=69116)		SMD
**Characteristics**	**n**	**%**	**n**	**%**		**n**	**%**	**n**	**%**	
≤2	439513	8.50	36246	62.31	1.36	115653	57.34	28993	57.49	0.003
3-5	702791	13.60	15325	26.34	0.32	59517	29.51	14833	29.41	0.002
>5	4026926	77.90	6601	11.35	1.8	26538	13.16	6601	13.09	0.002
Mean ± SD	9.87±5.14		2.47±2.75		1.79	2.71±2.94		2.69±2.89		0.008
**Sex**										
Female	2504475	48.45	25741	44.25	0.08	89350	44.30	22422	44.46	0.003
Male	2664755	51.55	32431	55.75	0.08	112358	55.70	28005	55.54	0.003
**Comorbidity**										
Allergic rhinitis	18179	0.35	464	0.80	0.06	817	0.41	408	0.81	0.05
Urticaria	14567	0.28	389	0.67	0.06	547	0.27	323	0.64	0.05
Atopic dermatitis	53073	1.03	9712	16.70	0.57	9151	4.54	1967	3.90	0.03
**Urbanization**										
1 (high)	1474890	28.53	15974	27.46	0.02	55460	27.50	14018	27.80	0.007
2	1560935	30.20	18239	31.35	0.03	65780	32.61	15876	31.48	0.02
3	991819	19.19	10428	17.93	0.03	35238	17.47	9136	18.12	0.01
4 (low)	1141586	22.08	13531	23.26	0.03	45230	22.42	11397	22.60	0.004
**Follow-up period, years**	7.48±4.00		7.02±4.02		0.11	7.48±4.08		7.03±4.03		0.11

Data shown as n(%) or mean ± SD. SMD, standardized mean difference. A standardized mean difference of ≤ 0.10 indicates a negligible difference between the two groups.

**Table 2:** The incidence rate of KD in different factors.

Variables	Kawasaki disease (n=373)			Crude HR (95% CI)	Adjusted HR (95% CI)
	Event	PY	IR		
**Non-typhoidal *Salmonella***					
No	281	1510777	1.86	1(reference)	1(reference)
Yes	92	354963	2.59	1.33(1.05-1.68)**	1.31(1.03-1.66)**
**Age, years**					
≤2	328	1075141	3.05	1(reference)	1(reference)
3-5	43	552953	0.78	0.25(0.18-0.35)***	0.24(0.18-0.34)***
>5	2	237645	0.08	0.02(0.007-0.10)***	0.03(0.006-0.10)***
**Sex**					
Female	158	828894	1.91	1(reference)	1(reference)
Male	215	1036845	2.07	1.08(0.88-1.33)	1.12(0.92-1.38)
**Comorbidities**					
Allergic rhinitis					
No	371	1859816	1.99	1(reference)	1(reference)
Yes	2	5924	3.38	1.20(0.30-4.82)	1.99(0.54-6.12)
Urticaria					
No	373	1860827	2.00	1(reference)	1(reference)
Yes	0	4913	0.00	--	--
Atopic dermatitis					
No	364	1793933	2.03	1(reference)	1(reference)
Yes	9	71807	1.25	0.55(0.28-1.07)	1.05(0.58-2.68)
Urbanization					
1 (high)	118	504863	2.34	1(reference)	1(reference)
2	102	589395	1.73	0.74(0.57-1.02)	0.76(0.58-1.03)
3	66	335101	1.97	0.87(0.64-1.17)	0.86(0.63-1.16)
4 (low)	87	436380	1.99	0.89(0.67-1.17)	0.90(0.68-1.19)

**p < 0.01, ***p < 0.001.

PY, person-years; IR, incidence rate, per 10,000 person-years; HR, hazard ratio; CI, confidence interval; HR adjusted for age, sex, allergic rhinitis, urticarial, atopic dermatitis and urbanization.

**Table 3:** Subgroup analysis.

Variables	NTS infection						Crude HR(95% CI)	Adjusted HR (95% CI)	p for interaction
	No			Yes					
	Event	PY	IR	Event	PY	IR			
Total	281	1510777	1.86	92	354963	2.59	1.33(1.05-1.68)**	1.31(1.03-1.66)**	
**Age, years**									0.97
≤2	247	863934	2.86	81	211206	3.84	1.31(1.02-1.68)*	1.31(1.02-1.69)*	
3-5	32	453043	0.71	11	99910	1.10	1.43(0.72-2.83)	1.45(0.73-2.88)	
>5	2	193799	0.10	0	43845	0.00	--	--	
**Sex **									0.48
Female	116	671523	1.73	42	157371	2.67	1.47(1.03-2.08)*	1.46(1.03-2.08)*	
Male	165	839253	1.97	50	197591	2.53	1.23(0.90-1.69)	1.21(0.88-1.66)	
**Comorbidities**									
Allergic rhinitis									0.79
No	280	1507072	1.86	91	352743	2.58	1.32(1.05-1.68)*	1.32(1.04-1.67)*	
Yes	1	3704	2.70	1	2219	4.51	1.76(0.11-28.29)	3.12(0.16-38.56)	
Urticaria									0.99
No	281	1507936	1.86	92	352890	2.61	1.33(1.05-1.69)*	1.31(1.04-1.66)*	
Yes	0	2840	0.00	0	2073	0.00	--	--	
Atopic dermatitis									0.42
No	275	1451590	1.89	89	342342	2.60	1.30(1.02-1.65)*	1.31(1.03-1.67)*	
Yes	6	59186	1.01	3	12620	2.38	2.32(0.58-9.26)	2.51(0.61-10.35)	
**Urbanization**									0.96
1 (high)	89	406387	2.19	29	98476	2.94	1.30(0.86-1.98)	1.31(0.86-1.99)	
2	79	479862	1.65	23	109533	2.10	1.21(0.76-1.93)	1.17(0.73-1.86)	
3	49	270010	1.81	17	65090	2.61	1.37(0.78-2.37)	1.39(0.80-2.42)	
4 (low)	64	354517	1.81	23	81862	2.81	1.45(0.90-2.34)	1.42(0.89-2.32)	
**Total**	281	1510777	1.86	92	354963	2.59	1.33(1.05-1.68)**	1.31(1.03-1.66)**	

*p < 0.05, **p < 0.01.

PY, person-years; IR, incidence rate, per 10,000 person-years; HR, hazard ratio; CI, confidence interval; HR adjusted for age, sex, allergic rhinitis, urticarial, atopic dermatitis and urbanization.

**Table 4:** Sensitivity analysis (Model 2).

Variables	Model 1 + KD coded by pediatric specialists (n=313)			Crude HR (95% CI)	Adjusted HR(95% CI)
	Event	PY	IR		
**NTS infection**					
No	239	1511144	1.58	1(reference)	1(reference)
Yes	74	355103	2.08	1.26(0.98-1.64)	1.35(0.96-1.62)

PY, person-years; IR, incidence rate, per 10,000 person-years; HR, hazard ratio; CI, confidence interval; HR adjusted for age, sex, allergic rhinitis, urticarial, atopic dermatitis and urbanization.

**Table 5:** Sensitivity analysis (Model 3).

Variables	Model 2 + KD coded in the Registry for Catastrophic Illness Patient Database (n=98)			Crude HR (95% CI)	Adjusted HR(95% CI)
	Event	PY	IR		
**Nontyphoidal Salmonellosis**					
No	71	1512137	0.47	1(reference)	1(reference)
Yes	27	355434	0.76	1.55(1.01-2.41)*	1.54(1.00-2.40)*

*p < 0.05.

PY, person-years; IR, incidence rate, per 10,000 person-years; HR, hazard ratio; CI, confidence interval; HR adjusted for age, sex, allergic rhinitis, urticarial, atopic dermatitis and urbanization.

We tracked all patient records for every subject in the two groups from their enrollment date until the first diagnosis of KD, the date they turned 18 years old, withdrawal from the National Health Insurance Program, or the end of 2013 if they were free of KD. We adjusted demographic variables, including age at index date, sex, urbanization, and comorbidities, to minimize potential confounding effects.

Allergic diseases (20, 21), including asthma, were recognized as comorbidities associated with the development of KD in this study. To avoid the confounding effect of asthma, we excluded all the patients who had a diagnosis of asthma (ICD-9-CM 493) before the outcome date (when they had the first diagnosis of KD, the date they turned 18 years old, or the end of the study). Other common allergic diseases were matched and analyzed in our study, including preexisting allergic rhinitis (ICD-9-CM 477.x), urticaria (ICD-9-CM 708.x), and atopic dermatitis (ICD-9-CM 691.x).

Furthermore, the risk of developing KD was stratified according to covariates including demographic characteristics, comorbidities, and different levels of urbanization.

### Statistical Analysis

We performed Chi square (χ2) tests to determine homogeneity among categorical variables, including age group, sex, comorbidities and different levels of urbanization, between the nontyphoidal salmonellosis (NTS) study group and the non-NTS group. The Kaplan-Meier method with log rank test were used to demonstrate the cumulative incidence of KD among the two groups. We calculated the incidence rates of KD per 10,000 person-years in both groups. Additionally, to investigate the association between NTS infection and subsequent KD, we conducted Cox proportional regression models to estimate hazard ratios (HRs) and 95% confidence intervals (CIs) after adjusting for relevant covariates.

Apart from the main model, for the purpose of validation, we conducted a series of sensitivity analyses, which included model 2: KD patients who also had ICD-9-CM coded by pediatric specialists; and model 3: KD patients with diagnosis on the Registry for Catastrophic Illness Patient Database. The sensitivity analyses were implemented to evaluate the HR of KD with presence of NTS. A further analysis with an alternative outcome was conducted (**Table 6**), to determine the relationship between NTS and the risk of Henoch-Schönlein purpura (a falsification end point), which is another common vasculitis among children. From literature review, Henoch-Schönlein purpura is associated with previous upper respiratory infection rather than NTS. If there is association between NTS and Henoch-Schönlein purpura, there might be unmeasured confounding. On the contrary, if there is no association between NTS and Henoch-Schönlein purpura using our study model, the unmeasured confounding might be small from the point of view. The data and statistics were processed and analyzed by SAS^®^ (version 9.4; SAS Institute, Inc., Cary, NC, USA), and a P-value of < 0.05 was considered to indicate statistical significance.

**Table 6:** Alternative outcome analysis.

Variables	Henoch–Schönlein purpura (n=189)			Crude HR (95% CI)	Adjusted HR(95% CI)
	Event	PY	IR		
**NTS infection**					
No	151	1553622	0.97	1(reference)	1(reference)
Yes	38	366264	1.04	1.05(0.74-1.50)	1.04(0.73-1.49)

PY, person-years; IR, incidence rate, per 10,000 person-years; HR, hazard ratio; CI, confidence interval; HR adjusted for age, sex, allergic rhinitis, urticarial, atopic dermatitis and urbanization.

## Results

In this study, we enrolled 69,116 patients with nontyphoidal salmonellosis (NTS) from January 1st, 2000, to December 31^st^, 2013; 276,464 patients without NTS were matched (at a 1:4 ratio) by propensity score. As demonstrated in **Table 1**, the two cohorts shared similar baseline characteristics, including age, sex, comorbidities, and level of urbanization. The mean ages of the NTS group and non-NTS group were 2.69 ± 2.89 and 2.71 ± 2.94, respectively, with follow-up periods of 7.03 ± 4.03 and 7.48 ± 4.08 years, respectively.

**Figure 1:**
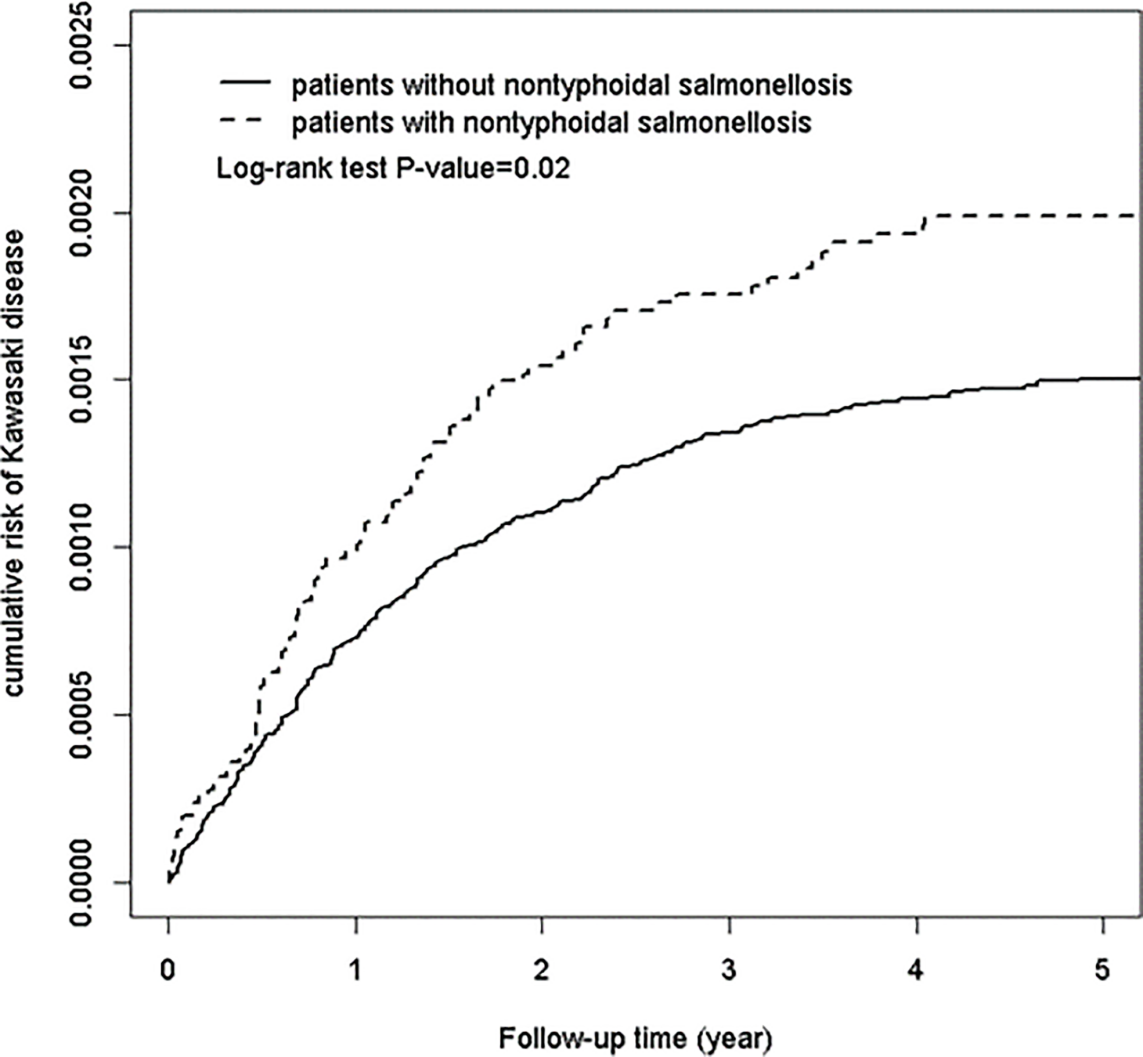
Cumulative risk of Kawasaki disease in patients with and without non-typhoidal *Salmonella* infection.

During the follow-up period, Kaplan-Meier analysis showed that patients with NTS had a significantly higher cumulative incidence of KD than the non-NTS group (log-rank test, p < 0.0001), as depicted in **Figure 1**. The incidence rates of KD in the NTS and non-NTS groups were 2.59 and 1.86 per 10,000 person-years, respectively. To examine the assumption of constant proportionality, we used scaled Schoenfeld residuals. In the follow-up period, the assumption of proportional hazard was met (P-value = 0.68).

**Table 2** demonstrates the results of multivariable analysis of covariates associated with KD, including demographic characteristics and comorbidities. The patients with NTS had a crude hazard ratio [HR] of 1.33 (95% confidence interval [CI] = 1.05-1.68; p < 0.01) of KD compared with the non-NTS cohort. After adjusting for the associated covariates listed in **Table 1**, NTS (adjusted hazard ratio [aHR] = 1.31; 95% CI = 1.03-1.66; p < 0.01) was an independent risk factor for KD. Additionally, compared with older age subgroups, those aged ≤2 years had the highest risk of developing KD. The crude HRs of the ≤2, 3-5, and >5 age groups were 1 (reference), 0.25 (95% CI, 0.18-0.35), and 0.02 (95% CI, 0.007-0.10), respectively. Additionally, the male group tended to have a risk of developing KD (aHR =1.12; 95% CI, 0.92-1.38), though the difference was not significant. There were no other significant risk factors identified among other comorbidities and covariates.

The results were robust in serial sensitivity analyses. In the main model (**Tables 1**–**3**), the adjusted HR was 1.31 (95% CI, 1.03-1.66), with KD events defined as patients with at least two clinic visits or one admission that were registered with ICD-9-CM code 446.1. In model 2 (**Table 4**), the adjusted HR was 1.35 (95% CI, 0.96-1.62), with KD patients defined as also having ICD-9-CM codes for pediatric diagnosis. In model 3 (**Table 5**), the adjusted HR was 1.54 (95% CI, 1.00-2.40), with KD patients identified also on the Registry for Catastrophic Illness Patient Database.

**Table 3** depicts the risk of KD associated with patient characteristics, comorbidities, and other covariates. In the subgroup analysis stratified by age, NTS was significantly associated with a higher risk of KD in children aged ≤2 years (aHR= 1.31, 95% C.I. 1.02-1.69). In sex subgroup analysis, a significantly higher risk of KD was found in female patients (aHR= 1.46, 95% C.I. 1.03-2.08) with NTS. The effect of NTS was not significant in either of the urbanization subgroups. Regarding comorbidity subgroup analysis, higher risks of KD were observed in subjects without allergic rhinitis, urticaria, and atopic dermatitis.

**Table 6** revealed the incidence and hazard ratio of Henoch-Schönlein purpura (alternative outcome analysis) in NTS and non-NTS group. The risk of Henoch-Schönlein purpura was not significantly higher in patients with NTS compared with those without NTS.

## Discussion

To our knowledge, this is the first study to investigate the epidemiologic association between NTS and KD. The results showed a 1.31-fold higher risk, in patients with NTS infection.

Although the origin of KD is not fully understood, infection has long been suspected as an etiology, largely based on its multisystemic clinical presentation and epidemiological characteristics (22). A variety of mechanisms have been proposed to be involved in systemic vasculitis related to infections, such as molecular imitation, superantigen, Toll-like receptor (TLR) activation, and antineutrophil cytoplasmic antibody (ANCA)-associated responses (23, 24). There have been a variety of bacterial and viral pathogens reported in KD, though there is no strong evidence for their roles in pathogenesis (25, 26). After decades of searching, including through microbiological, molecular and serological techniques, no etiological agent of KD has been successfully identified until now.

At first, some other microbiological methods were used in attempts to isolate pathogens from body fluids and animal inoculation of these specimens (27), and molecular techniques to detect circulating conserved microbial nucleic acid sequences in KD patients also failed (28). However, it has been demonstrated in some animal models that environmental factors might play a role in the etiology by modulating the risk of infection, whereby coronary arteritis was induced in animal models injected with microbial components from Lactobacillus and Candida (29, 30). A later hypothesis is as follows: superantigens, which may be related to bacterial toxins, trigger the cascade that leads to KD. This is based on the evidence that KD has clinical, pathological, immunological similarities, and even occasional concurrence in children with toxic shock syndrome, which is superantigen mediated (31, 32). Superantigens bind to the Vbeta2 region of the T-cell receptor, and clonal expansion of Vbeta2-expressing T cells has been recognized in some studies; nonetheless, findings are inconsistent, and the theory is widely debated (33). A prospective study demonstrated that the isolation rates of superantigen-producing bacteria between patients with KD and febrile controls were not statistically significant, though a difference in isolation rates of bacteria producing Vbeta2-stimulatory toxins was detected (34). Another study found an oligoclonal IgA-directed immune response in the vascular wall in acute KD and supported an Ag-driven immune response, suggesting a conventional antigen in the pathogenesis of KD (35).

To help identify the exact causative agent, innovative molecular techniques may be required. On the other hand, it is reasonable to investigate infections arising from zoonotic reservoirs, which are also capable of spreading systemically, in exploration of possible associations with KD. Non-typhoidal *Salmonella* is one of the leading bacterial pathogens of foodborne infections, both in developed and developing regions, and it often occurs in infants and elderly individuals (36). Acute gastroenteritis associated with nontyphoidal *Salmonella* infection is characterized by a short period of incubation, generally less than 1 day, with manifestations of diarrhea, fever, and neutrophil-dominant intestinal inflammatory infiltrates (37, 38). The zoonotic pathogens *Salmonella enterica* serotype Typhimurium and *S. enterica* serotype Enteritidis are two of the most frequently associated pathogens (39). *Salmonella* bacteria invade the gastrointestinal tract and submucosal lymphoid system and then enter and survive within macrophages (40). These bacteria can elude immunological surveillance and remain for a long time within a host (41). Their ability to survive and replicate within macrophages facilitates their dissemination. *Salmonella* Typhimurium, for example, is likely to disseminate systemically, reaching important organs (42). NTS infections occur mainly in infants and children (43), and the susceptibility of young children and infants to NTS might be explained by gut immaturity, gastric hypoacidity and transmission within families (44).

In our subgroup analysis stratified by age, NTS was significantly associated with a higher risk of KD in children aged ≤2 years. This association was not significant in the older age groups (3-5 years and >5 years); however, there were scarce cases of KD in the older age groups both in the NTS cohort and the non-NTS cohort. As for sex subgroup analysis, the effect of NTS was significant in females, with no subgroup differences. In addition, the relationship of KD and urbanization is not clearly determined in this study, as our results indicated no significantly higher risk of KD in either of the urbanization subgroups with NTS. However, there was a slight tendency for the group among NTS patients with lower urbanization to have a higher incidence of KD, though the P-value for interaction in the urbanization subgroup was not significant.

A numbers of risk factors for NTS infections in infants have been recognized, including exposure to reptiles, meat or poultry, consumption of concentrated liquid infant formula, traveling abroad, and attending day care where children have diarrhea (45). In Taiwan, however, the specific predisposing factors for children toward NTS infections have not yet been identified due to the lack of associated ecological studies. The study conducted by P. L. Chen et al. demonstrated a peak of NTS infection during summer, and this seasonal variation may be explained by human behaviors, the virulence of pathogens influenced by the environment, and variations in host immunity (46).

There had been number of studies exploring the relationship between allergic diseases and KD (20, 21). However, our results were not consistent with the previous findings, as the scarcity of cases of KD in patients with allergic rhinitis, urticaria, and atopic dermatitis, might have to some extent biased the results.

Based on the recognition that NTS and KD share very similar epidemiological distributions, including the major age groups, high-prevalence regions, and consistency in their seasonality, we further identified NTS as an independent risk factor for KD. However, more prospective studies are needed to determine the mechanism explaining the relationship between infection, allergies, ecological factors, humoral response, and systemic vasculitis in KD.

A specific strength of this study is that we used a nationwide population-based data set, which may have provided a sufficient sample size to explore the epidemiologic association between NTS and KD. Additionally, we controlled for confounding factors from other comorbidities to minimize bias. Nonetheless, we are aware of several limitations in our study to be addressed. First, NTS and KD diagnoses based on administrative claims data recorded by physicians and hospitals may be less accurate than those made on a clinical basis and in a prospective setting. For example, misclassification bias might occur due to underdiagnosis or overdiagnosis. However, as the NTS patients we identified were mostly according to positive stool cultures, the results are reliable despite being underestimated. Additionally, since the Taiwan NHI assesses accuracy in medical coding and claims validity in medical charts, it has been reported that the NHI Research Database provides acceptable validity for epidemiologic research. Second, additional patient data, including body mass index, ethnic group, lifestyle, family history, and environmental factors, were not available in the database and thus were not included in our analysis. Third, NTS severity was not provided in the database. To reduce the potential confounding effects caused by the factors above, we adjusted the data analysis for components such as age, sex, associated comorbidities, and level of urbanization.

In conclusion, our study found that NTS is an independent risk factor for KD in Taiwanese children. As many risk factors for salmonellosis were identified in young children, who are also the target group of KD, we further call for more prospective studies to explore the relationship between NTS infection and development KD.

## Data Availability Statement

The original contributions presented in the study are included in the article/supplementary material. Further inquiries can be directed to the corresponding authors.

## Author Contributions

All authors contributed to the article and approved the submitted version. Conception and design. RC, Y-MH, J-NL, JW. Acquisition of data. L-TC, J-NL. Analysis and interpretation of data. Y-MH, TC, M-CC, RC, L-TC, J-NL, JW. Writing (original draft preparation): Y-MH, TC, M-CC, L-TC. Writing (review and editing): Y-MH, JW.

## Funding

This study was supported in part by China Medical University Hospital (DMR-110-222) and Kaohsiung Veterans General Hospital (VGHKS107156). The funders had no role in the design and conduct of the study; the collection, management, analysis, and interpretation of the data; or the preparation, review, or approval of the manuscript.

## Conflict of Interest

The authors declare that the research was conducted in the absence of any commercial or financial relationships that could be construed as a potential conflict of interest.
